# Supplementary material for: A high-resolution mRNA expression time course of embryonic development in zebrafish
Source: eLife. 2017 Nov 16;6:e30860. doi: 10.7554/eLife.30860 (PMC5690287; doi:10.7554/eLife.30860)
Supplement: Supplementary file 6. [file elife-30860-supp6.zip › biolayout-clusters-files/Cluster101-genes.html]

Cluster101


# Cluster101: Genes

| Ensembl ID | Gene Name | Chr | Start | End | Biotype |
| --- | --- | --- | --- | --- | --- |
| ENSDARG00000090861 | CABZ01087514.1 | 3 | 60367547 | 60379564 | protein\_coding |
| ENSDARG00000036473 | ENSDARG00000036473 | 7 | 25844946 | 25853913 | protein\_coding |
| ENSDARG00000089564 | MDFI | 11 | 21949319 | 22005273 | protein\_coding |
| ENSDARG00000057708 | PHTF1 | 11 | 18639960 | 18656836 | protein\_coding |
| ENSDARG00000101143 | VPS37B (1 of many) | 10 | 45057064 | 45068310 | protein\_coding |
| ENSDARG00000011466 | lace1a | 17 | 6608391 | 6632440 | protein\_coding |
| ENSDARG00000010169 | myd88 | 24 | 20044495 | 20048577 | protein\_coding |
| ENSDARG00000007181 | nadk2 | 21 | 22293348 | 22310098 | protein\_coding |
| ENSDARG00000027813 | nus1 | 20 | 42324048 | 42344346 | protein\_coding |
| ENSDARG00000015474 | ppp2r5ea | 20 | 20331965 | 20370518 | protein\_coding |
| ENSDARG00000019660 | rxfp2b | 15 | 31877952 | 31914678 | protein\_coding |
| ENSDARG00000068152 | si:dkey-77f17.1 | 18 | 26697182 | 26732778 | protein\_coding |
| ENSDARG00000004055 | uhrf1bp1l | 4 | 18218860 | 18292110 | protein\_coding |
